# Genome Sequence of PM2-Like Phage Cr39582, Induced from a Pseudoalteromonas sp. Isolated from the Gut of Ciona robusta

**DOI:** 10.1128/genomeA.00368-18

**Published:** 2018-05-24

**Authors:** Brittany A. Leigh, Mya Breitbart, Hanna M. Oksanen, Dennis H. Bamford, Larry J. Dishaw

**Affiliations:** a College of Marine Science, University of South Florida, St. Petersburg, Florida, USA; b Department of Pediatrics, Children’s Research Institute, University of South Florida, St. Petersburg, Florida, USA; c Molecular and Integrative Research Programme, Faculty of Biological and Environmental Sciences, University of Helsinki, Helsinki, Finland

## Abstract

Phage Cr39582 was induced by mitomycin C from Pseudoalteromonas sp. strain Cr6751, isolated from a marine invertebrate gut. Pseudoalteromonas phage Cr39582 has 85% pairwise nucleotide identity with phage PM2 but lacks sequence homology in the spike protein. This report supports previous bioinformatic identification of corticoviral sequences within aquatic bacterial genomes.

## GENOME ANNOUNCEMENT

Pseudoalteromonas virus PM2 is currently the only virus species of the Corticoviridae family (1). The classification is based on the presence of an internal membrane layer and a supercoiled circular double-stranded DNA (dsDNA) genome of approximately 10 kb (2, 3). PM2 is a virulent marine phage discovered off the coast of Chile in the 1960s (2, 4). Although sequences related to phage PM2 are widespread within the genomes of aquatic proteobacteria (5), it is unknown if these prophages can be experimentally induced (5). To date, seven genomes of virulent phages infecting Pseudoalteromonas strains and 31 prophages have been reported (6). Here, we describe the genome sequence of Pseudoalteromonas phage Cr39582, a prophage of Pseudoalteromonas sp. strain Cr6751 that contains a circular dsDNA genome of 10,584 bp with 20 predicted open reading frames (ORFs). Phage Cr39582 is only the second described PM2-like phage and a potential member of the family Corticoviridae; thus, its genome sequence increases our understanding of nontailed dsDNA viruses that may represent an abundant and ecologically important fraction of marine viral communities (7, 8).

Pseudoalteromonas sp. Cr6751 was isolated from the gut of the marine invertebrate Ciona robusta, as described previously (9). Bacterial identity was determined based on full-length 16S rRNA gene Sanger sequencing. The presence of active prophages was determined based on exposure of the bacterial culture to 0.5 µg ml^−1^ mitomycin C (10). The culture supernatant was passed through a 0.22-μm filter, and the filtrate was stained with SYBR Gold to enumerate viral-like particles (VLPs) (11). The VLPs were purified by cesium chloride density gradient ultracentrifugation (1.23 g ml^−1^) and imaged by transmission electron microscopy (TEM), revealing a nontailed viral particle (∼45-nm capsid diameter) resembling phage PM2. DNA was extracted from the purified filtrate using the Qiagen MinElute virus spin kit and sequenced on the Illumina MiSeq platform (2 × 250 bp). The resulting 71,931 reads were trimmed with Trimmomatic version 0.35.0 and assembled with SPAdes version 3.6.0 according to the iVirus pipeline (12). The assembled genome had 3,038-fold coverage and a G+C content of 48.2%.

ORFs were called using GLIMMER3 (13) and annotated using a BLASTx search against the GenBank nonredundant database. The genome of phage Cr39582 is syntenous with phage PM2, demonstrating sequence homology throughout the genome with the exception of the ORF spanning positions 7982 to 9430, which is most similar to hypothetical Pseudoalteromonas sp. proteins. This region corresponds to the PM2 gene encoding the spike protein, which recognizes a host receptor that results in fusion with the bacterial membrane, ultimately allowing genome entry (14, 15). Phage Cr39582 was not able to form plaques on phage PM2’s bacterial host, Pseudoalteromonas espejiana BAL-31, likely due to the dissimilarity between the two spike proteins. Full-genome alignment of phage Cr39582 to PM2 revealed ∼85% overall identity. Pairwise nucleotide identities between Cr39582 and PM2 in the 5′ and 3′ regions flanking the spike protein gene were ∼91% and ∼96%, respectively.

### Accession number(s).

The phage Cr39582 genome sequence and the 16S rRNA gene sequence of the host Pseudoalteromonas sp. Cr6751 were deposited in GenBank under accession numbers MG966533 and MH014962, respectively.
